# An automatic segmentation for improved visualization of atrial ablation lesions using magnetic resonance imaging

**DOI:** 10.1186/1532-429X-13-S1-P251

**Published:** 2011-02-02

**Authors:** Rashed Karim, Aruna Arujuna, Jaspal Gill, Mark ONeill, Jaswinder Gill, Reza Razavi, Daniel Rueckert, Tobias Schaeffter, Kawal Rhode

**Affiliations:** 1 King's College London, London, UK; 2 Guy's and St Thomas' NHS Foundation Trust, London, UK; 3 Imperial College London, London, UK

## Background

Delayed-enhancement (DE) MRI is an effective technique for imaging left atrial (LA) ablation lesions following radio-frequency ablation for atrial fibrillation (AF). Existing techniques for segmentation/visualization of lesions require manual interaction of an expert user making them prone to high observer variability. Oakes *et al.*[1] applied thresholding on the intensity histogram of the manually-outlined atrial wall. Peters *et al.*[2] used maximum intensity projections (MIPs) to generate volume lesion visualizations for expert-user interpretation. Knowles *et al.*[3] employed MIP followed by user-interactive thresholding for lesion surface visualization.

## Purpose

The aim of our work was to develop a fully-automated approach for LA lesion segmentation/visualization.

## Methods

Five patients with paroxysmal AF (2 male, average age 55 years, LA size 4.2±0.5cm) underwent pulmonary vein (PV) isolation achieving successful electrical isolation of all PVs. Imaging was performed 6-months post-ablation on a Philips 1.5T Achieva with scans including respiratory-navigated and cardiac-gated whole-heart 3D-SSFP and inversion recovery-prepared MRI for visualization of Gd-DTPA DE (complete LA coverage, resolution of 1.3×1.3×2mm^3^).

The endocardial cavity of the LA was segmented from the whole heart SSFP-MRI scan. The segmented LA was registered to the DE-MRI scan using DICOM header data. The atrial wall was approximated by a ±3mm thick region from the endocardial surface. For each voxel in the wall, the probability of it being labelled as scar or healthy was derived from trained classifiers. For the healthy-tissue class, a mixture of Gaussian distributions was used to model the observed atrial wall tissue, with parameters computed using the Expectation-Maximization algorithm. For the scar-tissue class, the classifier was trained on *prior* segmentations. The DE-MRI images were segmented using the proposed algorithm and by an expert using the approach in [3].

## Results

**Figure 1:**
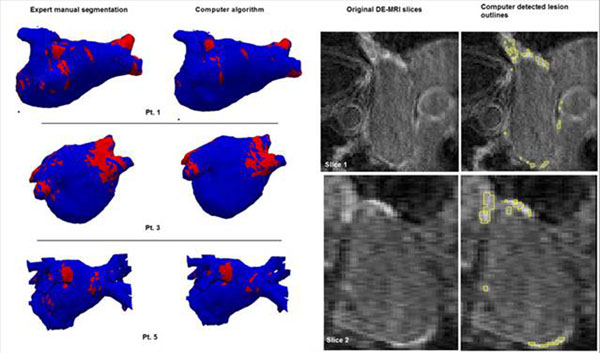
(left image) Surface visualization of 3 cases (patients 1, 3 and 5 from Table 1) with red regions indicating detected areas of scar. (right image) 3D voxel-wise segmentation of scar as seen on two selected slices from patient 1.

The algorithm segmented scars in less than 30 seconds on a 2.2GHz PC with *no* user interaction. The total surface area of scar was computed and represented as a percentage of the atrial surface area. Table 1 and Figure 1 show that there was good agreement between the results from the novel approach and the expert’s semi-automatic segmentation.

**Table 1:** The number of cardiac surface vertices classified into the healthy and scar tissue categories based on semi-automated and automatic segmentation. There was no statistical difference in the methods (p=0.36 paired t-test).

	Automated algorithm (# surface vertices)		Expert-operated semi-automated (# surface vertices)		% scar using automated algorithm	% scar using expert-operated semi-automated	% difference
Patient	Healthy	Scar	Healthy	Scar			

Pt. 1	4937	1167	5196	13.38	18.24	13.38	+4.86
Pt. 2	7953	260	7954	2.87	2.77	2.87	-0.10
Pt. 3	6555	629	6540	8.31	8.67	8.31	+0.36
Pt. 4	6354	1584	6220	25.08	23.00	25.08	-1.92
Pt. 5	4522	602	4500	12.00	15.50	12.00	-3.50

## Conclusions

This study demonstrates a fully-automatic and rapid segmentation/visualization of post-ablation LA DE-MRI that will minimise the observer variability that is seen with existing approaches that require expert manual interactions.

## References

[B1] Oakes Circulation 2009 119 13 1758 67 10.1161/CIRCULATIONAHA.108.811877 19307477 PMC2725019

[B2] Peters Radiology 2007 243 3 690 5 10.1148/radiol.2433060417 17517928

[B3] Knowles IEEE Trans 2010 57 6 1467 75 10.1109/TBME.2009.2038791 20172807

